# Past injurious exercise attenuates activation of primary calcium‐dependent injury pathways in skeletal muscle during subsequent exercise

**DOI:** 10.14814/phy2.13660

**Published:** 2018-03-29

**Authors:** Ryo Takagi, Riki Ogasawara, Junya Takegaki, Yuki Tamura, Arata Tsutaki, Koichi Nakazato, Naokata Ishii

**Affiliations:** ^1^ Graduate School of Health and Sport Science Nippon Sport Science University Tokyo Japan; ^2^ Department of Life Sciences The University of Tokyo Tokyo Japan; ^3^ Department of Life Science and Applied Chemistry Nagoya Institute of Technology Aichi Japan

**Keywords:** Calpain, contraction, JNK, mechanical stress, repeated bout effect

## Abstract

Past contraction‐induced skeletal muscle injury reduces the degree of subsequent injury; this phenomenon is called the “repeated bout effect (RBE).” This study addresses the mechanisms underlying the RBE, focusing on primary calcium‐dependent injury pathways. Wistar rats were subdivided into single injury (SI) and repeated injury (RI) groups. At age 10 weeks, the right gastrocnemius muscle in each rat in the RI group was subjected to strenuous eccentric contractions (ECs). Subsequently, mild ECs were imposed on the same muscle of each rat at 14 weeks of age in both groups. One day after the exercise, the RI group showed a lower strength deficit than did the SI group, and neither group manifested any increase in membrane permeability. The concentration of protein carbonyls and activation of total calpain increased after ECs given at the age of 14 weeks. Nonetheless, these increases were lower in the RI group than in the SI group. Furthermore, calcium‐dependent autolysis of calpain‐1 and calpain‐3 in the RI group was diminished as compared with that in the SI group. Although peak ankle joint torque and total force generation during ECs at the age of 14 weeks were similar between the two groups, phosphorylation of JNK (Thr^183^/Tyr^185^), an indicator of mechanical stress applied to a muscle, was lower in the RI group than in the SI group. These findings suggest that activation of the primary calcium‐dependent injury pathways is attenuated by past injurious exercise, and mechanical stress applied to muscle fibers during ECs may decrease in the RBE.

## Introduction

Excessive exercise causes skeletal muscle injury, especially after eccentric contractions (ECs) rather than concentric or isometric contractions (Howatson and van Someren 2008; Nosaka 2011). The injury induces a prolonged muscle strength deficit; furthermore, muscle fibers can undergo necrosis in case of severe injury (Kano et al. 2008). As a skeletal muscle adaptation to the injury, injured skeletal muscle reduces the severity of the symptoms of subsequent muscle injury, a phenomenon called the “repeated bout effect” (RBE) (Nosaka et al. 2001; Stupka et al. 2001; Vissing et al. 2008). McHugh et al. (1999) have suggested that complex adaptations in neurons, connective tissue, and muscle fibers are involved in the RBE. Furthermore, Hyldahl et al. (2017) have reported that neural adaptations, alterations to muscle mechanical properties, structural remodeling of the extracellular matrix (ECM), and biochemical signaling are involved in the potential mechanism of the RBE; however, the details are unknown.

In pathways of contraction‐induced injury, extracellular calcium influx through stretch‐activated channels (Yeung et al. 2005; Zhang et al. 2012) elevates intracellular calcium levels (Sonobe et al. 2008). The upregulated calcium activates calpain (Khorchid and Ikura 2002; Murphy et al. 2007) and phospholipase (Ryan et al. 2000), and may enhance reactive oxygen species (ROS) formation (Grijalba et al. 1999; Ott et al. 2002). Studies suggest that calpain activation and ROS are related to the strength deficit in contraction‐induced muscle injury (Badalamente and Stracher 2000; Moopanar and Allen 2005). Additionally, calpain and phospholipase activation events and lipid peroxidation by ROS increase membrane permeability (Duncan and Jackson 1987; Mason et al. 1997; Zhang et al. 2008), and the secondary extracellular calcium influx can induce muscle fiber necrosis in severe cases (Ownby et al. 1978).

Recently, we developed an animal model of the RBE, which is suitable for clarifying adaptations in connective tissue and muscle fibers (excluding neurons) due to controlled electrical stimulation and showed that past injurious exercise causes collagen deposition and fiber type conversion (Takagi et al. 2016), which are associated with a reduction in muscle injury (Hyldahl et al. 2015; Takagi et al. 2016). Mechanical forces are transmitted between a cell and its surrounding ECM (Roca‐Cusachs et al. 2013). An increase in the levels of *α*7*β*1 integrin, which links the ECM with the cellular cytoskeleton, inhibits signaling pathways associated with muscle injury (Boppart et al. 2006). In particular, JNK phosphorylation, which highly correlates with the tension applied to a muscle during ECs (Martineau and Gardiner 2001), decreases (Boppart et al. 2006). Therefore, collagen deposition may also reduce the mechanical stress applied to muscle fibers and can attenuate the primary elevation of intracellular calcium levels. Additionally, calcium uptake during contractions is lower in a slow muscle than in a fast muscle (Gissel and Clausen 1999), and a fast‐twitch fiber is more susceptible to injury than a slow one (Lieber and Fridén 1988; Lieber et al. 1991). On the basis of these data, we hypothesized that past injurious exercise attenuates activation of primary calcium‐dependent injury pathways in the RBE.

In this study, models of EC‐induced necrotizing and non‐necrotizing injuries were set up to investigate the influence of past injurious exercise [that causes collagen deposition and fiber type conversion (Takagi et al. 2016)] on activation of primary calcium‐dependent injury pathways after subsequent injurious exercise.

## Methods

### Animals

Fifty‐four 10‐week‐old male Wistar rats (CLEA Japan, Tokyo, Japan) were maintained in a 12:12 h light–dark cycle and allowed ad libitum access to food and water throughout the experiments. This study's protocol was approved by the ethical committee of the Nippon Sport Science University (approval No. 014‐A03).

### The experimental procedure

The rats were randomly assigned to one of two groups: single injury (SI) or repeated injury (RI). The right gastrocnemius muscle of the RI group was subjected to strenuous ECs at the age of 10 weeks as reported previously (Takagi et al. 2016). Then, the rats in both groups were subjected to mild ECs (described below) at 14 weeks of age. The 4 weeks interval between two successive bouts of ECs in the RI group was employed because isometric strength completely recovers 4 weeks after the injury (Takagi et al. 2016). Animals in both groups were euthanized by a combination of anesthesia and cervical spine fracture prior to or 15 min, 6 h (*n* = 7, respectively), or 1 days (*n* = 6) after ECs at the age of 14 weeks after measurement of ankle joint torque as described elsewhere (Takagi et al. 2016). Samples of the right medial gastrocnemius muscle belly were divided for histological and biochemical analyses, rapidly frozen in liquid nitrogen, and stored at −80°C.

### Models of EC‐induced necrotizing or non‐necrotizing muscle injury

The models of EC‐induced necrotizing and non‐necrotizing injuries were based on muscle injuries at the age of 10 and 14 weeks, respectively. The rats were anesthetized with isoflurane and firmly fixed on a custom‐made isokinetic dynamometer platform in the prone position. The right gastrocnemius muscle was electrically stimulated with electrodes attached to the muscle belly and the Achilles's tendon percutaneously. Stimulation voltage was set to achieve maximal twitch torque, and tetanic stimulation was given for 0.3 and 2.0 sec with a train of 4 msec rectangular pulses at 10 msec intervals in the models of necrotizing and non‐necrotizing injuries, respectively. At the onset of the electric stimulation, the ankle joint was isokinetically dorsiflexed to cause an EC. The speed and range of the forced lengthening were 180 and 30° per second and from 60 to 125° and 60 to 120° of the ankle joint angle in the models of necrotizing and non‐necrotizing injuries, respectively. ECs in the necrotizing injury model consisted of 60 tetanic contractions at 2 sec intervals. On the other hand, ECs that cause non‐necrotizing injury consisted of five sets of 10 tetanic contractions at 8 sec intervals with a 3 min interval between the sets. Edema and limping were visible only immediately after the ECs. Thus, the rats were not given postprocedure pain relief medication because interventions that may affect regeneration after injury should be excluded.

### Measurement of ankle joint torque

Isometric tetanic torque was measured as described previously (Song et al. 2004). Stimulus intensity was adjusted to produce the maximal isometric twitch force. Isometric planter‐flexion torque of the right ankle joint was measured with a dynamometer at a joint angle of 90°.

### Histological analysis

The Evans Blue dye (EBD) was used to detect partial necrosis of the muscle fibers (Matsuda et al. 1995; Barbier et al. 2004). EBD can enter only muscle fibers with a damaged membrane (Barbier et al. 2004; Lovering et al. 2007). Immediately after ECs at the age of 14 weeks, EBD (Sigma, St. Louis, MO, USA) in sterile phosphate‐buffered saline (PBS) was intraperitoneally injected into the rats at a volume of 1% of the body mass (1 mg of EBD in 0.1 mL of PBS per 10 g of body mass).

### Biochemical analysis

Western blotting was performed as reported elsewhere (Ogasawara et al. 2014; Takagi et al. 2016) with slight modifications to measure calpain‐1, calpain‐3, and total and phospho‐JNK (Thr^183^/Tyr^185^) levels. For the detection of calpain‐1 and calpain‐3, another homogenizing buffer was applied according to another report (Kanzaki et al. 2014). Muscle samples for other detection assays were homogenized in RIPA buffer (Thermo Scientific, Waltham, USA) containing a protease inhibitor (Roche Applied Science, Upper Bavaria, Germany) and a phosphatase inhibitor (Thermo Scientific). The homogenates were centrifuged at 20,000*g* for 15 min at 4°C. Twenty micrograms of protein was separated by sodium dodecyl sulfate‐polyacrylamide gel electrophoresis (SDS‐PAGE) and then transferred to a membrane. The membranes were blocked with 5% powdered milk or bovine serum albumin in Tris‐buffered saline containing 0.1% Tween 20 for 1 h at room temperature and incubated overnight at 4°C with a primary antibody. Primary antibodies against calpain‐1 (cat. #C0355; Sigma), calpain‐3 (cat. #NCL‐CALP‐12A2; Novocastra Laboratories, Newcastle upon Tyne, UK), total c‐Jun N‐terminal kinase (JNK) (cat. #9252; Cell Signaling Technology, Danvers, MA, USA), or phospho‐JNK (Thr^183^/Tyr^185^, cat. #9251; Cell Signaling Technology) were employed. The membranes were next incubated for 1 h at room temperature with an appropriate secondary antibody. Chemiluminescent reagents (Thermo Scientific) served for signal detection. Images were captured with Ez‐capture (ATTO, Tokyo, Japan), and the signals were quantified in the CS analyzer software (ATTO). After image capture, membranes were stained with Coomassie Brilliant Blue to normalize the signal intensities to the amount of each protein loaded, in accordance with another study (Welinder and Ekblad 2011).

Protein carbonyl content was measured with a detection kit (cat. #ROIK03; SHIMA Laboratories, Tokyo, Japan). Samples were prepared as described above. The membrane after the transfer was reacted with 2,4‐dinitrophenylhydrazine, and the protein‐bound 2,4‐dinitrophenylhydrazone was detected with an antidinitrophenyl antibody.

Total calpain activity was measured by means of the Calpain Activity Assay kit (cat. #ab65308; Abcam plc, Cambridge, UK). Frozen muscle samples were subjected to this analysis.

### Statistics

**Figure 1:**
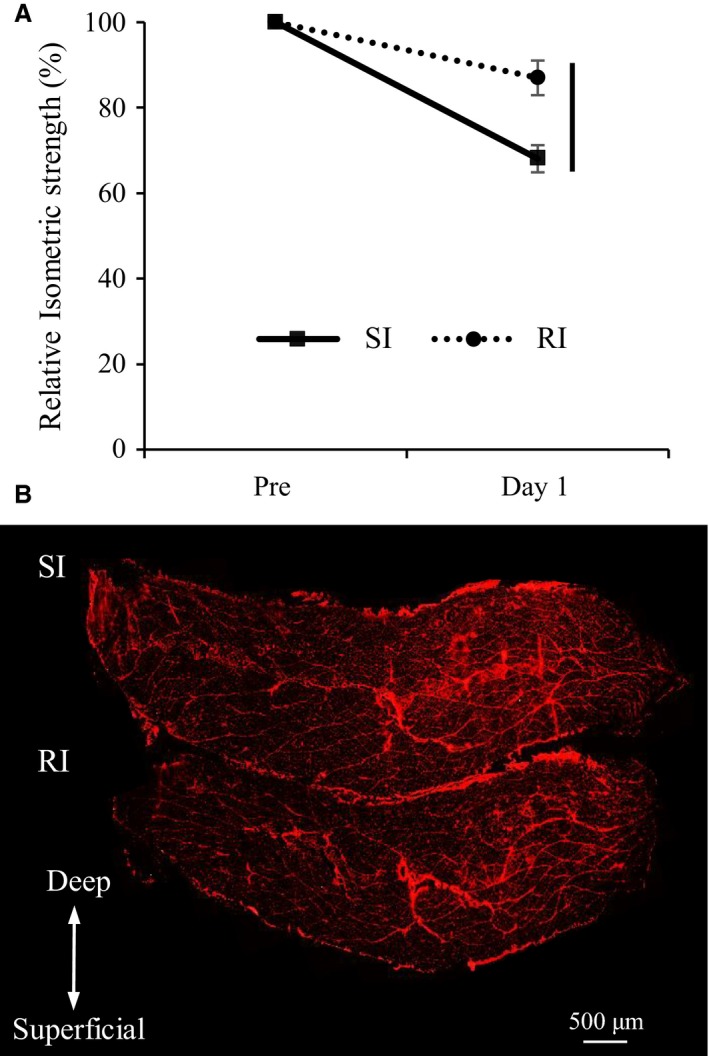
Changes in isometric strength (A) and representative photographs of an EBD‐stained (red) whole‐muscle cross section 1 days after ECs at 14 weeks of age (B). Maximal isometric torque values are expressed as a ratio of that measured after the ECs (day 1) to that measured before the ECs (Pre). Values are presented as means ± standard deviations. The vertical line means a significant difference between groups SI and RI at the same time point (*P* < 0.05). SI, single‐injury group; RI, repeated injury group; and EBD, Evans blue dye.

Data are expressed as means ± standard deviation (SD). Differences between the two groups were determined by the Welch *t* tests (Figs. 1A, 4A and D). The differences in the proportion of EBD‐positive fibers were determined by one‐way analysis of variance, and the other differences were examined by a two‐way analysis of variance followed by Bonferroni's post hoc test. Data with *P* < 0.05 were considered statistically significant.

## Results

### The strength deficit and EBD‐positive fibers after ECs at the age of 14 weeks

Isometric strengths measured 1 days after ECs at the age of 14 weeks decreased to 68.2 ± 2.3% and 87.0 ± 4.0% in groups SI and RI, respectively, as compared to strength measurements prior to the ECs (Fig. 1A). The deficit was significantly lower in the RI group than in the SI group. Figure 1B shows representative photomicrographs acquired 1 days after the ECs. Both groups SI (0.13 ± 0.04%) and RI (0.13 ± 0.02%) showed the same percentage of EBD‐positive fibers relative to total number of muscle fibers as in the intact muscle (0.09 ± 0.04%). These findings suggest that past injurious exercise reduces the strength deficit during the subsequent exercise that does not substantially increase membrane permeability.

### Protein carbonyls

**Figure 2:**
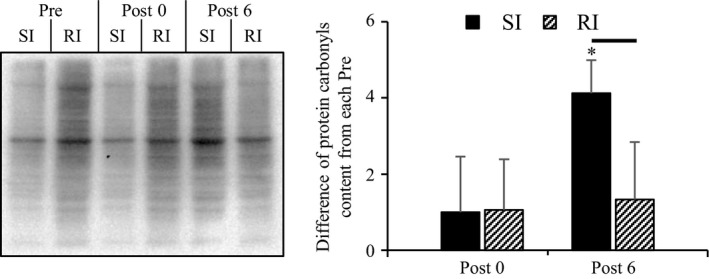
Differences in protein carbonyl content at Post 0 and Post 6 from the measurement before ECs in each group. Representative bands of protein carbonyl in each group are shown on the left. Values are presented as means ± standard deviations. * As compared to Post 0. The line between the two bars means a significant difference between groups SI and RI at the same time point (*P* < 0.05). SI, single‐injury group; RI, repeated injury group; Post 0, at 15 min after ECs; and Post 6, at 6 h after ECs.

The levels of protein carbonyls served as a marker of oxidative stress (Dalle‐Donne et al. 2003). The differences in protein carbonyl content between time points Post 0 or Post 6 and Pre were determined in each group. The SI group at Post 6 showed a higher value than that at Post 0 and the corresponding values in the RI group (Fig. 2). This finding suggested that past injurious exercise reduces protein carbonyl content during the subsequent exercise.

### Total calpain activity

**Figure 3:**
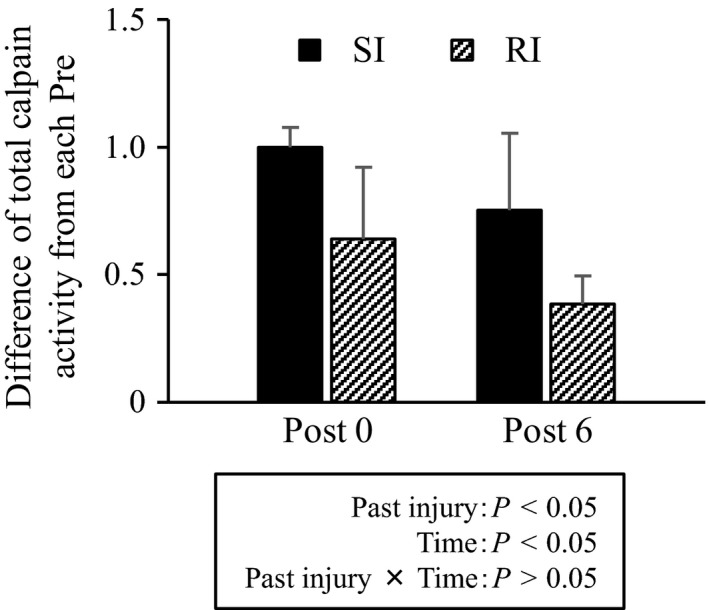
The difference in total calpain activity at Post 0 and Post 6 from the measurement before ECs in each group. Values are presented as means ± standard deviations. SI, single‐injury group; RI, repeated injury group; Post 0, at 15 min after ECs; and Post 6, at 6 h after ECs.

No interactions were associated with the difference in total calpain activity at Post 0 and Post 6 from each Pre; however, the RI group showed a lower average value than the SI group did, and Post 6 showed a lower average than Post 0 did (Fig. 3). This finding suggested that past injurious exercise attenuates total calpain activation during the subsequent exercise.

### Calpain‐1 and ‐3 autolysis

**Figure 4:**
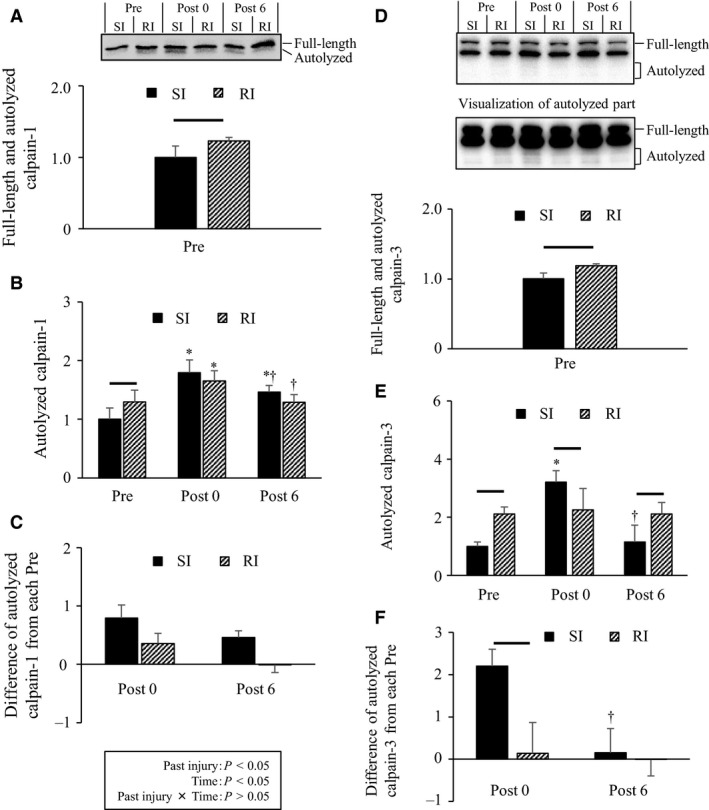
Total calpain‐1 and ‐3 content (A and D): a time course showing the changes in the levels of autolyzed‐calpain‐1 and ‐3 (B and E), and the increase in autolyzed‐calpain‐1 and ‐3 amounts at Post 0 and Post 6 relative to the measurement before ECs in each experimental group (C and F). Representative blots in each group appear at the top. Values are presented as means ± standard deviations. * As compared to Pre, ^†^ as compared to Post 0. The line between the two bars means a significant difference between groups SI and RI at the same time point (*P* < 0.05). SI, single‐injury group; RI, repeated injury group; Post 0, at 15 min after ECs; and Post 6, at 6 h after ECs.

Calpain undergoes autolysis in a calcium‐dependent manner (Murphy et al. 2006). The RI group showed higher total calpain‐1 content than the SI group did prior to ECs at the age of 14 weeks (Fig. 4A). Autolyzed‐calpain‐1 content can be ranked in the following order: Pre < Post 6 < Post 0 in the SI group, and Pre and Post 6 < Post 0 in the RI group (Fig. 4B). Additionally, the RI group showed higher autolyzed‐calpain‐1 levels than the SI group did prior to ECs at the age of 14 weeks. As for the increase relative to each level before ECs, the RI group showed a lower average than the SI group did (Fig. 4C). The RI group showed higher total calpain‐3 content than the SI group prior to ECs at the age of 14 weeks (Fig. 4D).As for autolyzed‐calpain‐3, the SI group showed a higher value at Post 0 than at Pre or Post 6 or RI group at Post 0, whereas the RI group showed no changes with time and higher values at Pre and Post 6 than the SI group did (Fig. 4E). As for the increase from each level before ECs, the SI group showed a higher value at Post 0 than the RI group did (Fig. 4F). These findings suggested that past injurious exercise reduced calpain‐1 and ‐3 autolysis during subsequent exercise despite the increase in total calpain‐1 and ‐3 levels.

### Mechanical parameters with muscle contractions

No significant differences were observed between the two groups in peak ankle joint torque (295.3 ± 38.8 mN·m in the SI group and 285.8 ± 32.1 mN·m in the RI group) and total force generation (7.27 ± 0.42 N·m·sec in the SI group and 7.31 ± 0.74 N·m·sec in the RI group) during ECs at the age of 14 weeks. These findings suggest that the mechanical stress imposed on the injured muscle was similar to that in an intact muscle.

### Phosphorylation of JNK (Thr^183^/Tyr^185^)

**Figure 5:**
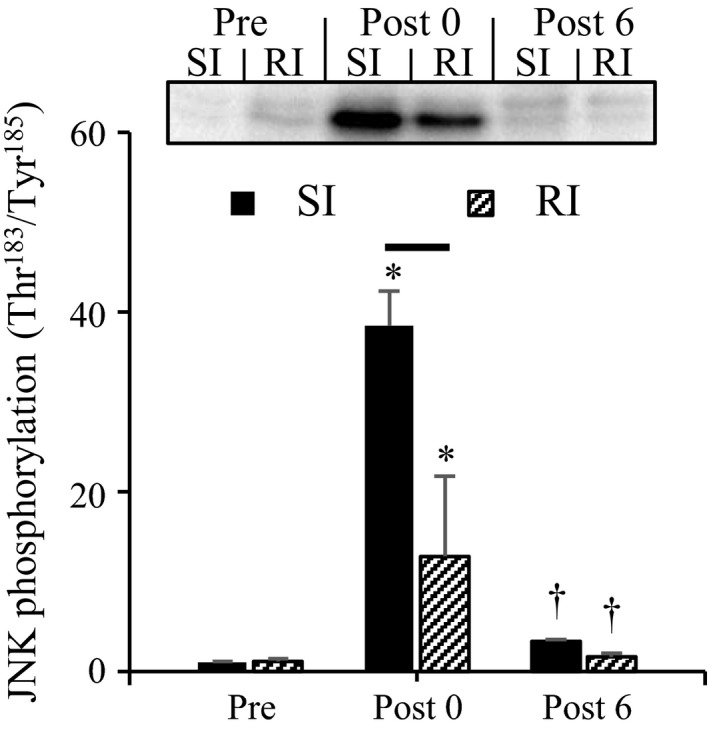
Time course of the changes in phospho‐JNK (Thr^183^/Tyr^185^). Representative blots in each group appear on the top. Values are presented as means ± standard deviations. *versus Pre, ^†^versus Post 0. The line between the two bars means significant difference between SI and RI at the same time point (*P* < 0.05). SI, single‐injury group; RI, repeated injuries group; Post 0, at 15 min after ECs; and Post 6, at 6 h after ECs.

Both groups showed higher levels of phospho‐JNK **(**Thr^183^/Tyr^185^) at Post 0 than at Pre and Post 6, but the SI group showed a higher value than the RI group did at Post 0 (Fig. 5). There was no difference in total JNK content between the groups prior to ECs at the age of 14 weeks (data not shown).

## Discussion

In this study, we investigated the influence of past injurious exercise on activation of primary calcium‐dependent injury pathways during subsequent exercise. Past injurious exercise attenuated calpain activation and protein carbonyl levels in the pathways. In conjunction with this mechanism, mechanical stress applied to muscle fibers may decrease, and consequently primary elevation in intracellular calcium levels may be attenuated.

Excessive exercise activates calcium‐dependent injury pathways, results in a prolonged muscle strength deficit, and may induce necrosis of muscle fibers in severe cases (Kano et al. 2008). As for the injury at the age of 10 weeks in the RI group, we have previously shown that about 10% of muscle fibers undergo necrosis (Takagi et al. 2018). Additionally, isometric strength completely recovers, and collagen deposition and partial fiber type conversion are detectable prior to the exercise at the age of 14 weeks (Takagi et al. 2016) under the same conditions as in this study. Here, past injurious exercise reduced the strength deficit and neither group showed a remarkable increase in membrane permeability after the exercise at the age of 14 weeks. Damaged membrane induces subsequent extracellular calcium influx; therefore, the injury model at the age of 14 weeks can detect the changes in activation of primary calcium‐dependent injury pathways.

Among the calcium‐dependent injury pathways, calpain activation and ROS are involved in the strength deficit (Badalamente and Stracher 2000; Moopanar and Allen 2005). Calpain‐3 is thought to have a role in the damage to excitation‐contraction coupling (Verburg et al. 2005). In the RI group, total calpain activation and protein carbonyl content were increased by ECs at the age of 14 weeks and were lower than those in the SI group. These findings suggest that past injurious exercise attenuates calpain activation and ROS‐induced injury during the subsequent exercise, resulting in a reduction in the strength deficit.

Calpain autolysis, which is thought to be necessary for calpain activation (Diaz et al. 2004) proceeds in a calcium‐dependent manner (Murphy et al. 2006). Because it is difficult to measure in vivo intracellular calcium levels of the gastrocnemius muscle, calpain autolysis was investigated to estimate these levels. In this study, the increase in calpain‐1 and calpain‐3 autolysis after ECs at the age of 14 weeks was lower in the RI group than in the SI group despite the increase in total calpain‐1 and ‐3 contents. These findings suggest that past injurious exercise attenuates primary elevation in intracellular calcium levels during the subsequent exercise, resulting in a reduction in calpain activation and of ROS‐induced injury.

Regarding the increased levels of autolysis of calpain‐1 and ‐3 at rest in the RI group, increased calpain‐3 autolysis is observed even on day 28 of muscle regeneration after nerve injury (Wu et al. 2015). In addition, calpain‐3 is thought to participate in sarcomere remodeling during muscle regeneration (Hauerslev et al. 2012). Activation of these calcium‐dependent pathways may be required for the later regeneration process although this notion is not yet proven.

We hypothesized that past injurious exercise attenuates activation of primary calcium‐dependent injury pathways after the subsequent exercise, especially owing to the reduction in mechanical stress on muscle fibers because of collagen deposition. Intracellular calcium levels are elevated via stretch‐activated channel‐induced extracellular calcium influx (Yeung et al. 2005; Sonobe et al. 2008; Zhang et al. 2012). In this study, the mechanical stress imposed on the entire muscle during the exercise at the age of 14 weeks was similar between the injured and intact muscles. Nevertheless, we have reported that an injured muscle under the same conditions as this study shows greater passive resistive torque than does an intact muscle (Takagi et al. 2016). Thus, it is possible that the mechanical stress imposed on the injured muscle is lower than that on the intact muscle at the fiber level. JNKs can be activated by various factors such as growth factors (Hibi et al. 1993), cytokines (Westwick et al. 1994), and stressors (Cano et al. 1994). Among them, JNKs phosphorylation is particularly responsive to mechanical stress during muscle contractions (Martineau and Gardiner 2001; Russ and Lovering 2006). Because it is difficult to measure the stress magnitude in vivo, JNK phosphorylation (Thr^183^/Tyr^185^) was evaluated to estimate the stress. The RI group showed lower phospho‐JNK (Thr^183^/Tyr^185^) levels than the SI group did immediately after ECs. Therefore, past injurious exercise may attenuate mechanical stress applied to muscle fibers during ECs, thereby contributing to attenuation of the elevation of intracellular calcium levels.

In conclusion, past injurious exercise attenuated activation of primary calcium‐dependent injury pathways during subsequent exercise. Partly because of this mechanism, mechanical stress applied to muscle fibers may decrease, and consequently primary elevation in intracellular calcium levels may be attenuated. Nevertheless, further research is needed on the mechanism underlying the attenuation of activation of calcium‐dependent injury pathways.

## Conflict of Interest

The authors declare no conflicts of interest and that the results of this study are presented clearly, honestly, and without fabrication, falsification, or inappropriate data manipulation. The results of this study do not constitute endorsement by the American Physiological Society.
